# Proteases of *Dermatophagoides pteronyssinus*

**DOI:** 10.3390/ijms18061204

**Published:** 2017-06-06

**Authors:** Thomas A. Randall, Robert E. London, Michael C. Fitzgerald, Geoffrey A. Mueller

**Affiliations:** 1 Integrative Bioinformatics Support Group, National Institute of Environmental Health Sciences, Research Triangle Park, NC 27709, USA; thomas.randall@nih.gov; 2 Genome Integrity and Structural Biology Laboratory, National Institute of Environmental Health Sciences, 111 T.W. Alexander Dr., Research Triangle Park, NC 27709, USA; london@niehs.nih.gov; 3 Chemistry Department, Duke Univeristy, Durham, NC 27708, USA; Michael.C.Fitzgerald@duke.edu

**Keywords:** allergens, proteases, allergen source-derived proteases, allergic sensitization, exposure

## Abstract

Since the discovery that Der p 1 is a cysteine protease, the role of proteolytic activity in allergic sensitization has been explored. There are many allergens with proteolytic activity; however, exposure from dust mites is not limited to allergens. In this paper, genomic, transcriptomic and proteomic data on *Dermatophagoides pteronyssinus* (DP) was mined for information regarding the complete degradome of this house dust mite. *D. pteronyssinus* has more proteases than the closely related Acari, *Dermatophagoides farinae* (DF) and *Sarcoptes scabiei* (SS). The group of proteases in *D. pteronyssinus* is found to be more highly transcribed than the norm for this species. The distribution of protease types is dominated by the cysteine proteases like Der p 1 that account for about half of protease transcription by abundance, and Der p 1 in particular accounts for 22% of the total protease transcripts. In an analysis of protease stability, the group of allergens (Der p 1, Der p 3, Der p 6, and Der p 9) is found to be more stable than the mean. It is also statistically demonstrated that the protease allergens are simultaneously more highly expressed and more stable than the group of *D. pteronyssinus* proteases being examined, consistent with common assumptions about allergens in general. There are several significant non-allergen outliers from the normal group of proteases with high expression and high stability that should be examined for IgE binding. This paper compiles the first holistic picture of the *D. pteronyssinus* degradome to which humans may be exposed.

## 1. Introduction

Proteases were initially recognized as promiscuous digestive enzymes that generally degraded other proteins; however, they are now known to perform a variety of other crucial biological processes. These include highly specific processing of signaling molecules in ovulation, fertilization, development, antigen presentation, inflammation and wound healing [1]. Proteases are generally classified on the basis of the mechanism of catalysis into five classes called aspartic, metallo, cysteine, serine and threonine [2]. Both aspartic- and metallo-proteases use an activated water molecule in a nucleophilic attack on the peptide bond. The latter three classes use the side chain of the named residue (Cys, Ser or Thr) as the source of the nucleophile. While all five classes of proteases are found in the human genome [1], according to the ALLFAM database of allergens, only aspartyl, cysteine and serine proteases have been identified as allergens [3].

Der p 1 was the first mite allergen to be characterized [4], and was eventually revealed to be a cysteine protease [5]. This immediately suggested a number of experiments to determine whether the catalytic activity of Der p 1 could influence allergic sensitization. For example, Der p 1 was shown to degrade endogenous protease inhibitors [6] and surfactant proteins [7], increasing the access of allergens to dendritic cells beneath the bronchial epithelial barrier. Proteases were also demonstrated to influence innate immune signaling via cleavage of the protease activated receptor, PAR-2 [8]. Downstream adaptive immune signaling can also be affected by protease activity. Der p 1 has been reported to target multiple proteins involved in the control of IgE synthesis [9,10,11]. It also has been pointed out that enzymatic activity of allergens could lead to undesirable effects in immunotherapies, suggesting that functional deactivation may be useful in this treatment [12]. Also regarding immunotherapy, the protease activity of the extract has been proposed as a useful quality control measure for extract standardization [13]. Understanding the mechanisms whereby an active protease may influence sensitization and desensitization is still an active area of research, as evidenced by this special issue of International Journal of Molecular Sciences (IJMS).

Since the identification of Der p 1 as a protease, many other proteases have been identified in other allergen sources, including cockroaches [14], fungi [15] and plants [16,17]. There are also three other mite allergens that are serine proteases: groups 3, 6 and 9 [18,19]. However, only Der p 1 is considered a major allergen [20], in that more than 50% of mite allergic patients react specifically to this protein with high IgE titres; recent studies showed the prevalence of Der p 1 reactivity is about 75% [21]. Human exposure related to symptoms and sensitization is thought to be primarily via mite fecal particles [4]. In a recent 2D gel analysis of DP feces, all four protease allergens could be detected, but Der p 3 and Der p 9 were less prominent [22]. However, allergens are also found prominently in whole mite extract, which is used in the diagnosis of mite allergy and in immunotherapy, hence it is also worthy of characterization for medical purposes [23].

Of course, humans are not exposed exclusively to mite allergens. In order to better characterize the allergens and non-allergens from dust mites to which humans are exposed, we previously have conducted genetic, transcriptomic and proteomic experiments on DP [24,25]. In this article, we have searched the DP genome [26] specifically for other proteases, and mined previous RNAseq and proteomic data for information regarding the potential proteases. Based on characteristics similar to known allergens, such as high abundance and high stability, these studies suggest that a number of other proteins from DP should be examined as candidate allergens, or for the potential to influence sensitization via protease activity. In addition, we present a comparative analysis of proteases from two closely related Acari for which genomic information is available, *Dermatophagoides farinae* (DF) and *Sarcoptes scabiei* (SS) [27,28].

## 2. Results

For the purposes of this study, a protease is defined by homology to the PROTIDENT database of known proteases [29], which is a curated list of the much larger and more encompassing MEROPS database [2]. PROTIDENT consists of 3051 proteases of all five classes, the sequences of which were downloaded from the PROTIDENT website (http://www.csbio.sjtu.edu.cn/bioinf/Protease/). Proteases were defined from the predicted proteome as those proteins having a BLASTP search match compared to PROTIDENT with an *E*-value less than 10^−5^. The lowest *E*-value match from the PROTIDENT protease database defined the type of protease for each of these 369 proteins meeting the *E*-value threshold above.

Table 1 shows the classification of *Dermatophagoides pteronyssinus* (DP) (369), *Dermatophagoides farinae* (DF) (267) and *Sarcoptes scabiei* (SS) (243) proteases. The distribution of protease groups is rather similar among these Acari and is also similar to *Homo sapiens*, where the aspartyl and threonine proteases are the least abundant, and the other groups are 5–10 times more frequent. For comparison, *Homo sapiens* have many more proteases, 553, as do *Mus musculus*, 628 [1].

**Figure 1:**
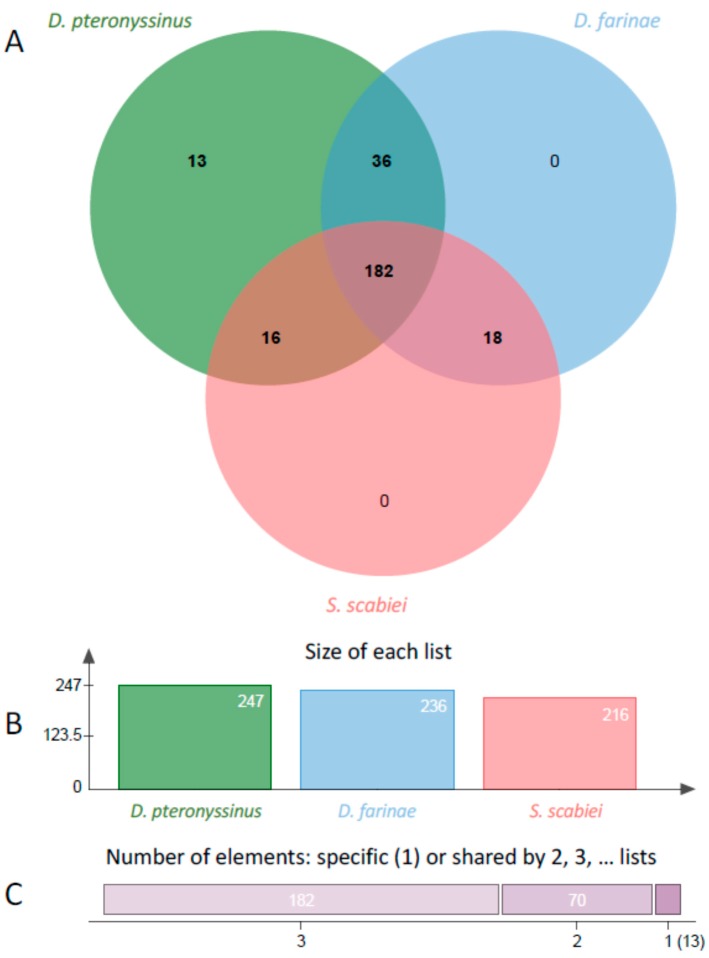
Venn diagram and statistics of proteases from three Acari. (**A**) Venn diagram of orthologous clusters of proteases from *D. pteronyssinus*, *D. farinae*, and *Sarcoptes scabiei*; (**B**) Total numbers of clusters in each species; and (**C**) Number of shared elements by number of groups.

The similarity of the Acari proteases is illustrated in Figure 1, which presents a Venn diagram of conserved orthologous clusters of the sets of proteases identified above, where a cluster is defined as a group of related proteins having a BLASTP score *E*-value less than 10^−5^ [30]. There were 247 clusters identified with DP having the most. Specifically, this means there are 247 proteases in DP that have at least one ortholog in either one or both of the other two species including 13 protease clusters of two or more related proteases unique to DP. There are 182 clusters containing at least one protease ortholog common to all three Acari. The total number of clusters is less than the total number of proteases (Table 1), because a given cluster can have more than one ortholog in any given cluster. However, DP had 13 clusters of proteases unrelated to proteins in DF or SS. Among these 13 clusters only four have a clear functional annotation, while the others are not well annotated functionally. None of these outliers are allergens, as the known allergens are closely related in homology.

**Figure 2:**
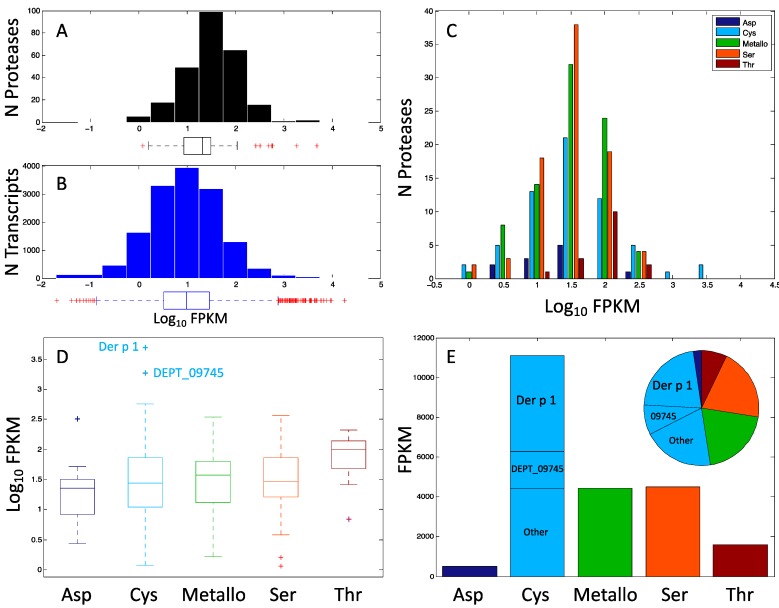
Statistical analyses of protease expression in *D. pteronyssinus*. (**A**) Histogram and boxplot of log_10_ fragments per kilobase per million reads (FPKM) (relative expression) of proteases in DP (plus signs indicate outliers from the quartile analysis); (**B**) Histogram and boxplot of log_10_ FPKM of all transcripts in DP; (**C**) Histogram of protease relative expression subdivided by protease type; (**D**) Boxplot of protease relative expression subdivided by type; and (**E**) Bar graph and inset pie chart of FPKM relative expression (not scaled by log_10_) colored according to protease type with scaled insets for Der p 1 and DEPT_09745.

One feature commonly reported for allergens is high abundance in the source. In order to assess the abundance of proteases on a genomic level, we reanalyzed our previously reported RNAseq data [25] to classify the expression of proteases in mite extract. Figure 2A shows a histogram of the fragments per kilobase per million reads (FPKM), or relative expression level of all the mite proteases as a histogram and box plot. For comparison, Figure 2B shows similar plots of all mite transcripts. The proteases appear to be distributed towards more highly expressed transcripts, and indeed a two-tailed *t*-test suggests that the two distributions are significantly different, *p* < 10^−4^, Table 2.

**Figure 3:**
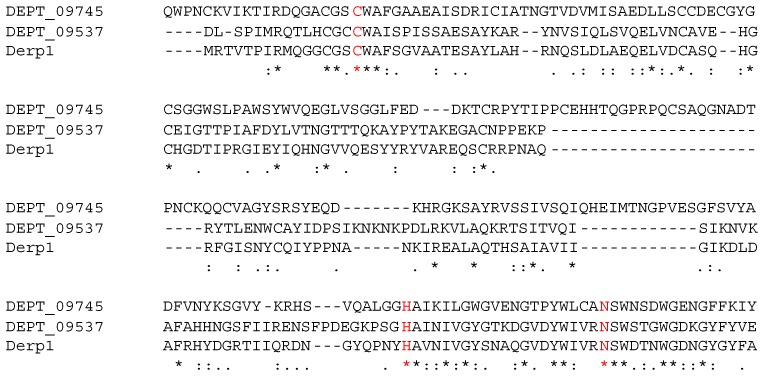
Alignment of catalytic residues of Der p 1 with two new C1 proteases. A multi-sequence alignment is shown for DEPT_09745, DEPT_09537 and Der p 1 over the region containing the Cys/His/Asn catalytic triad residues of Der p 1 in red (Cys34, His170, Asn190). “*”—Identity; “:”—strong similarity; “.”—similarity.

The FPKM values of the protease transcripts were further segregated according to the best match in the PROTIDENT database [29] (Figure 2C–E). Panel 2C shows that the most highly expressed proteases are in the cysteine family. The distributions of expression are rather similar, with the exception of the threonine proteases. The threonine protease expression distribution is slightly higher and narrower than all the others as shown by a *t*-test (*p* ≤ 0.05 for all comparisons, Table 2) and is shown visually in the boxplot analysis of panel 2D. The boxplot in panel 2D identifies that the most highly expressed cysteine protease is Der p 1, followed by a previously unknown protease DEPT_09745, which is only 22% identical to Der p 1. Figure 3 shows a partial alignment of Der p 1 with DEPT_09745 and DEPT_09537 (another C1 protease, see below) highlighting the catalytic triad residues Cys34, His170, and Asn190 of Der p 1. To better appreciate magnitude of expression levels in the mite, Panel E is plotted using raw FPKM values and not a log_10_ scaling as in the other panels. The bar graph shows that the sum of cysteine protease expression is the highest, and the inset pie chart demonstrates that Der p 1 accounts for 22% of protease expression in DP. In comparison, the sum total of Der p 3, Der p 6, and Der p 9 expression is only 3% of the total protease transcripts. The overabundance of cysteine protease expression may be related to the functional observation that extracts of DP and DF presented relatively higher cysteine protease activity than serine protease activity [12].

**Figure 4:**
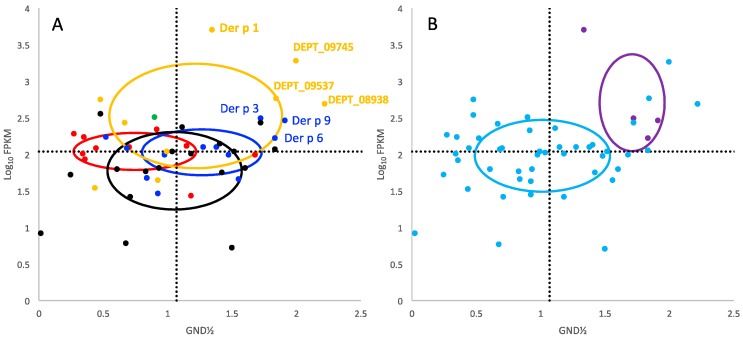
Stability and expression of DP proteases. Panels (**A**,**B**) are the same stability and expression data grouped differently. The colors in (**A**) refer to: green, aspartyl; yellow, cysteine; black, metallo; blue, serine; red, threonine proteases. The colors in (**B**) refer to: light blue, non-allergens, and purple, allergens. Dotted lines are the mean for the axis measurement of all proteases; *x*, GND½; *y*, log_10_ FPKM. Ellipses are centered at the mean in *x* and *y* of each group and the radii are determined by the standard deviations in *x* and *y* of each group.

Previously, we reported a combined analysis of transcription and protein stability for the allergens versus the non-allergens in DP and found that, as a group, the allergens were more abundantly expressed and more stable [25]. Stability was assessed for proteins in the mite extract using a combination mass spectrometry and guanidinium chloride denaturation approach termed Stability of Proteins from Rates of Oxidation (SPROX) [31,32]. The stabilities of the DP proteins were reported as the concentration of guanidinium chloride at the transition midpoint of the chemical denaturation curve (GND½), which is directly related to thermodynamic stability, see Methods. This protein abundance and stability data was parsed to segregate the proteases for analysis as shown in Figure 4. There were four allergens and 46 other proteases with both abundance and stability data. Panel 4A categorizes the proteases by type and Panel 4B categorizes the proteases by allergen versus non-allergen. The ellipses for each group are drawn with center at the mean and radii equal to the standard deviations in each respective measurement. The intent is to guide the readers’ eye, rather than to represent statistical significance.

For a rigorous statistical comparison, Hotelling’s *T*^2^ is a multidimensional *t*-test that was applied to test the variation in log_10_ FPKM and GND½ simultaneously, Table 3 [33]. Four *T*^2^ comparisons reached a *p*-value less than 0.05: cysteine versus metallo and serine proteases, serine versus threonine proteases, and the allergens versus non-allergens. However, only the allergen versus non-allergen comparison remains less than 0.05 using a Bonferroni corrected *p*-value. If we look at the *t*-tests for individual variables, for the variable GND½ the Bonferonni corrected *p*-value was less than 0.05 only for cysteine versus metallo-proteases. The Bonferonni corrected *p*-values are less than 0.05 only for the allergen versus non-allergen proteases for the variable FPKM, Table 2. This indicates that the FPKM variable likely dominated the multidimensional comparison in this case. As was concluded previously, being simultaneously highly expressed and highly stable increased the likelihood that a protein or protease was an allergen, but it was not predictive [25]. That is, there were numerous other proteases with similar measurements, as evidenced by the three cysteine proteases in Panel 4A with high transcript abundance and high stability.

A search of the Protein FAMily (PFAM) domain database found 273 non-peptidase domains in the 369 *D. pteronyssinus* peptidases discussed above. The most common domains associated with peptidase activity were alpha/beta hydrolases (129 domains of six subtypes), hemagglutinin repeats (50 domains of two subtypes) and ubiquitin carboxyl-terminal hydrolases domains (47 domains of two subtypes).

**Figure 5:**
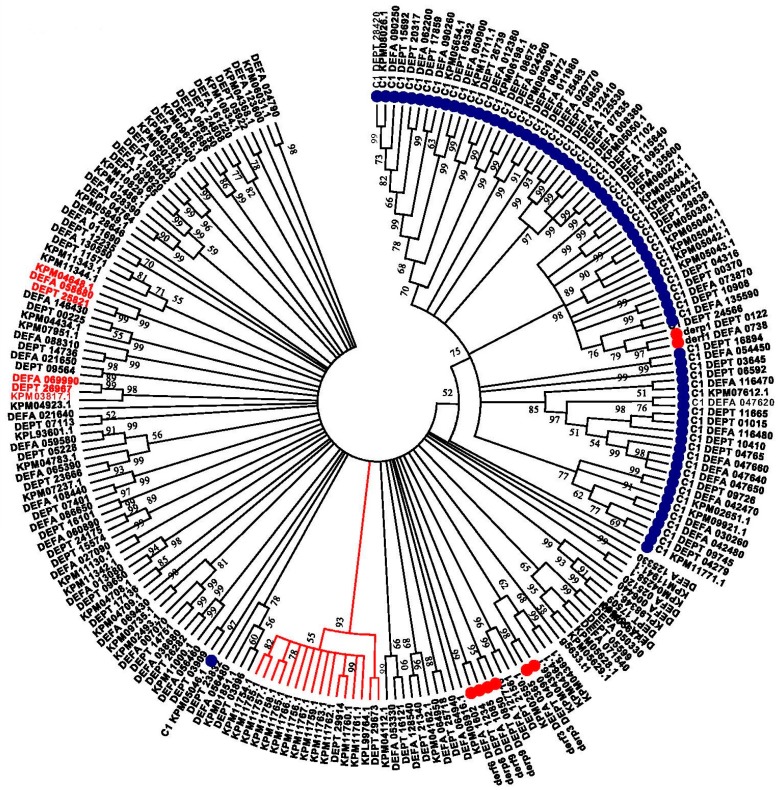
Phylogenetic tree of cysteine- and trypsin-related proteases within the Astigmata mites. A circular tree is depicted. 100 bootstrap iterations of the data were performed to estimate significance. All nodes with bootstrapping values >50 are shown. The branch lengths do not reflect the distances between the proteins in the tree but are drawn for visual clarity. The cysteine proteases are highlighted with a blue ball while all of the allergens are highlighted with red balls. The *S. scabiei* gene family expansion is noted by the branches highlighted in red. All of the proteases fitting the PFAM peptidase_C1 model are prefixed “C1”.

To focus on the proteases most closely related to the allergen proteases, we searched our predicted *D. pteronyssinus* proteome, and those of *D. farinae* and *S. scabiei*, with the appropriate PFAM Hidden Markhov Model (HMM) for these four allergens (see Methods). A phenogram of these proteins grouped the cysteine and trypsin domain-containing proteins separately, with one exception, and with good confidence; see Figure 5. Throughout the tree, virtually all of the proteins from each of the three species group into trios of orthologous proteins corresponding to proteins from the three species. These typically have strong bootstrapping support (two examples have protein names highlighted in red), although overall resolution between the orthologous trios of trypsin-related proteases shows less confidence. Occasionally, the ortholog trios showed a small expansion of one or two proteins in one of the species. One branch showed a significant expansion of a gene family in *S. scabiei* relative to *D. pteronyssinus*, with 14 paralogs versus two (DEPT_29673 and DEPT_29914), and no *D. farinae* ortholog, highlighted by the red line in Figure 5.

## 3. Discussion

It is commonly asserted that allergens are among the most abundant and stable proteins from a given allergy source. A reason for this may be that the proteins have to be hardy and/or abundant to survive the journey from the source to *Homo sapiens*. Recently, it was demonstrated that DP allergens as a group are simultaneously more highly expressed and more stable than the norm confirming this popular notion [25]. A number of caveats to this study should be mentioned which were technical compromises. The use of whole mite extract was designed to get maximum coverage of the mite proteome and transcriptome, but this may not completely represent natural human exposure to mites. Second, the use of transcription levels as a proxy for protein expression was again designed to sample as many genes as possible but there is not always a direct correlation. In a well-studied murine example [34], the correlation coefficient between transcription and protein levels was 0.4, indicating that the correlation is positive but imperfect. As a positive point, mites of all life stages were collected for analysis in this study, which is presumably representative of the natural environment. However, the diet in culture is known to affect the amount of allergen produced and this may or may not reflect the diet of mites in homes [35].

Figure 4 and Table 2 present strong statistical evidence that the protease allergens are simultaneously more highly expressed and more stable than other proteases in DP. It seemed appropriate to propose examining other mite allergens with similar properties for IgE reactivity because the list of known allergens may not necessarily be complete. Therefore, the three highly expressed cysteine proteases, and possibly the two metallo-proteases adjacent to Der p 3 and Der p 6 in Figure 4B in terms of stability and expression would be interesting candidates to investigate as potential allergens, since in Figure 4 they appear to have properties similar to other allergens. The three candidate cysteine proteases are DEPT_09537, DEPT_09745 and DEPT_08938. The first two are C1 proteases like Der p 1 (Figure 3), while the third is similar to NLPC_P60 cysteine proteases. The two candidate metallo-proteases are DEPT_09722 and DEPT_07233. However, to define a protein as an allergen the key question that remains to be answered is: do humans generate IgE antibodies to these proteins?

Further studies will be required to better characterize what other proteases are found in dust, and mite fecal particles. The genomic information will be extremely valuable in uniquely identifying proteins from mites using mass spectrometry. While not all of the proteases may be allergens, this study shows that mites harbor all five major categories of proteases. Therefore, studies that seek to understand the effect of proteases using mite extract should be cognizant of this and utilize other classes of proteases besides papain as a single surrogate for the cysteine protease Der p 1. Additionally, a cocktail of inhibitors will be required in control experiments that seek to inhibit the protease activity, since the different classes are blocked by different mechanisms. It may also be worthwhile to utilize various combinations and permutations of inhibitors if one seeks to isolate the class of proteases that could be most important in sensitization.

While this article has focused on DP proteases, it is worth commenting that many allergens are not proteases [36] and other factors have also been shown to affect sensitization. Examples include genetic susceptibility [37], the modern hygienic lifestyle [38], as well as natural and anthropogenic adjuvants [39,40]. Protease activity is likely one of many factors that can skew the immune response towards allergy.

## 4. Materials and Methods

Mite RNA and protein extract were collected and analyzed previously [24,25]. Briefly, DP was cultured at room temperature on Diet A [35]. Mites of all active life stages migrated onto the lids as food became depleted. Mites were collected by aspiration onto a 38 mm stainless steel mesh and were killed by freezing and then mites and spent culture were lyophilized. Aqueous mite extract was prepared from the mite material as described [41] in sterile Dulbecco’s Phosphate Buffered Saline without protease inhibitors and frozen prior to the analysis.

The transcriptome of *D. pteronyssinus* was assembled de novo from an RNAseq dataset derived from whole mite bodies and has been described previously [25]. The published proteome of *D. farinae* was obtained from Chan et al. [27]. The *S. scabiei* proteome is available from the Ensembl Metazoa Browser http://metazoa.ensembl.org/Sarcoptes_scabiei/Info/Annotation/ [42]. The predicted proteome of *D. pteronyssinus* is a combination of proteins derived from the transcriptome and two ab initio gene prediction to be described [26]. For the orthology analysis with OrthoVenn, amino acid sequences of the predicted proteases from each of the three mites outlined in Table 1 were input as separate fasta files (Supplemental) and the default threshold values were used (*E*-value 10^−5^ and inflation of 1.5) [30]. Additional domains within our *D. pteronyssinus* protease collection were determined using the HMMER3.1 [43] hmmscan function querying the complete PFAM domain database 27.0 [44,45]. For the phylogenetic analysis, cysteine- and trypsin-containing proteases were identified from the three mites of interest by searching each predicted proteome with two PFAM HMM models (Peptidase_C1; PF00112.21 and Trypsin; PF00089.24) and retrieving these sequences. These proteins were aligned with MAFFT [46] http://www.ebi.ac.uk/Tools/msa/mafft/ and input into MEGA 7.0 for phylogenetic analysis [47]. The relationships between proteins was inferred by using the Maximum Likelihood method based on the JTT matrix-based mode [48]. The tree with the highest log likelihood (−148,009.3839) is shown. Initial tree(s) for the heuristic search were obtained automatically by applying Neighbor-Join and BioNJ algorithms to a matrix of pairwise distances estimated using a JTT model, and then selecting the topology with superior log likelihood value. The analysis involved 211 amino acid sequences. There were a total of 4177 positions in the final dataset.

The protein stability data used in the statistical analyses described here were taken from reference [25]. The stability data was generated using the so-called “hybrid SPROX protocol” previously described [31]. Briefly, the mite extract containing a cocktail of protease inhibitors was distributed into a series of buffers containing increasing concentrations of the chemical denaturant, guanidinium chloride. The protein samples in the denaturant-containing buffers were reacted with hydrogen peroxide to selectively oxidize methionine residues and with dimethyl(2-hydrogen-5-nitrobenzyl)sulfonium bromide (HNSB) to selective modify tryptophan residues. The modification reactions were quenched, and the protein samples from each denaturant-containing buffer were submitted to a quantitative bottom-up proteomics analysis using an isobaric mass tagging strategy to evaluate the extent of methionine oxidation and tryptophan modification in the proteins from each chemical denaturant-containing buffer. Ultimately, the relative abundances of the wild-type (i.e., unmodified) methionine- and/or tryptophan-containing peptides derived from the proteins in the different denaturant-containing buffers were quantified, and the data were fit to a four-parameter sigmoidal equation using a nonlinear-least squares analysis to ultimately generate a GND½ value (i.e., the denaturant concentration at the midpoint of the unfolding transition).

Previously, it was noted that the addition of a protease inhibitor had a stabilizing effect on Der p 1 by 0.4 GND½ in the stability assay conducted [25]. Therefore, Der p 1, Der p 3, Der p 6, and Der p 9 were adjusted by −0.4 when making comparisons with non-allergens. Since a cocktail of protease inhibitors was added that should inhibit all classes, an adjustment to protease stability by −0.4 was included for all the non-allergen proteases identified in this study. See the supplemental information in [25] for a more detailed explanation of this correction factor.

Statistics were calculated in MATLAB (R2014a Mathworks). Hotelling’s $T^{2}$ is calculated according to

(1) $$T^{2}=n_{1}n_{2}{\left({\bar{x}}_{1}-{\bar{x}}_{2}\right)}^{'}C^{-1}\left({\bar{x}}_{1}-{\bar{x}}_{2}\right)/\left(n_{1}+n_{2}\right),$$

where the subscripts refer to the groups of data, n is the number of data points, x is the sample mean vector, and C is a pooled estimate of the covariance matrix calculated using the covariance matrices of the individual groups of data,

(2) $$C=\left\{\left(n_{1}-1\right)C_{1}+\left(n_{2}-1\right)C_{2}\right\}/\left(n_{1}+n_{2}-2\right).$$

Statistical significance can be estimated by transforming to an *F* statistic,

(3) $$F=\left(n_{1}+n_{2}-p-1\right)T^{2}/\left\{\left(n_{1}+n_{2}-2\right)p\right\}$$

where p is the number of variables, and the *F* distribution has p and $\left(n_{1}+n_{2}-p-1\right)$ degrees of freedom [33].

## 5. Conclusions

The *D. pteronyssinus* genome, transcriptome, and available stability data were surveyed to provide information on all the proteases from this species. This affords a better characterization of all the proteases to which humans could be exposed, either in the environment or as part of immunotherapy. Based on expression levels and stability data, several other DP proteases should be tested for IgE binding.

## Figures and Tables

**Table 1:** Number of proteases in related Acari.

Protease Type	*D. pteronyssinus*	*D. farinae*	*S. scabiei*
Threonine Proteases	18	20	17
Serine Proteases	133	88	76
Metalloproteases	127	94	83
Cysteine Proteases	77	60	61
Aspartyl Proteases	14	5	6
Total	369	267	243

**Table 2:** *t* Tests of fragments per kilobase per million reads (FPKM) and midpoint of guanidinium chloride denaturation curve (GND½).

Data Compared	Group 1	*n*_1_	Group 2	*n*_2_	*p* Value	Corrected *p* ^†^
log_10_ FPKM						
	all mite	14,406	proteases	255	<0.001	
	asp	11	cys	61	0.288	3.170
	asp	11	metallo	83	0.204	2.243
	asp	11	ser	84	0.172	1.891
	asp	11	thr	16	0.003	0.033
	cys	61	metallo	83	0.747	8.220
	cys	61	ser	84	0.783	8.612
	cys	61	thr	16	0.052	0.576
	metallo	83	ser	84	0.943	10.375
	metallo	83	thr	16	0.004	0.040
	ser	84	thr	16	0.003	0.029
*t* Tests FPKM and GND½ *						
GND½					
	cys	9	metallo	18	0.005	0.035
	cys	9	ser	12	0.043	0.301
	cys	9	thr	10	0.060	0.420
	metallo	18	ser	12	0.260	1.820
	metallo	18	thr	10	0.135	0.945
	ser	12	thr	10	0.178	1.246
log_10_ FPKM						
	allergens	4	non-allergens	45	0.010	0.070
	cys	9	metallo	18	0.490	3.430
	cys	9	ser	12	0.851	5.957
	cys	9	thr	10	0.092	0.644
	metallo	18	ser	12	0.266	1.862
	metallo	18	thr	10	0.135	0.945
	ser	12	thr	10	0.018	0.126
	allergens	4	non-allergens	45	0.007	0.049

* Note that Aspartyl proteases are not included because *n* = 1; ^†^ Bonferroni Corrected.

**Table 3:** *T*^2^ tests.

Data 1	Data 2	Group 1	Group 2	*T*^2^	*F*	df1	df2	*p*	Corrected *p* ^†^
GND½ *	log_10_ FPKM	cys	metallo	9.9	4.8	2	24	0.013	0.091
		cys	ser	6.9	3.2	2	18	0.047	0.329
		cys	thr	5.5	2.6	2	16	0.081	0.567
		metallo	ser	2.6	1.3	2	27	0.300	2.100
		metallo	thr	5.6	2.7	2	25	0.072	0.504
		ser	thr	6.8	3.2	2	19	0.046	0.322
		allergens	non-allergens	11.9	5.8	2	46	0.004	0.028

* Note that Aspartyl proteases are not included because *n* = 1; ^†^ Bonferroni Corrected; degrees of freedom (df).

## Supplementary Materials

Supplementary materials can be found at www.mdpi.com/1422-0067/18/6/1204/s1.

## Abbreviations

DP	*Dermatophagoides pteronyssinus*
DF	*Dermatophagoides farinae*
SS	*Sarcoptes scabiei*
GND½	Concentration of Guanidinium Chloride at the Transition Midpoint of the Chemical Denaturation Curve
SPROX	Stability of Proteins from Rates of Oxidation
FPKM	Fragments per Kilobase per Million Reads
